# Chronic Hematuria Increases Chronic Kidney Injury and Epithelial–Mesenchymal Transition in 5/6 Nephrectomy Rats

**DOI:** 10.3389/fmed.2021.753506

**Published:** 2021-11-25

**Authors:** Min Xiao, Ajay K. Medipally, Laura Biederman, Anjali A. Satoskar, Iouri Ivanov, Brad H. Rovin, Sergey V. Brodsky

**Affiliations:** ^1^Department of Pathology, The Ohio State University Wexner Medical Center, Columbus, OH, United States; ^2^Department of Pathology, Nationwide Children Hospital, Columbus, OH, United States; ^3^Department of Medicine, The Ohio State University Wexner Medical Center, Columbus, OH, United States

**Keywords:** hematuria, chronic kidney disease, anticoagulation, epithelial mesenchymal transition (EMT), iron

## Abstract

Chronic kidney disease (CKD) is a common outcome of many kidney diseases. Interstitial fibrosis and tubular atrophy (IFTA) is a histologic hallmark of CKD. Hematuria is a common symptom in many human kidney diseases. Free hemoglobin may affect tubular epithelial cells by generating reactive oxygen species (ROS). Epithelial–mesenchymal transition (EMT) of the tubular epithelial cells has been shown to play an important role in the IFTA development. The aim of this study was to determine the effects of chronic hematuria on the CKD progression in 5/6 nephrectomy (5/6NE) rat model of CKD. 5/6 NE rats were treated with oral warfarin (0.5 mg/kg/day) or vehicle (control). The animals were monitored for 26 weeks, while prothrombin time (PT), serum creatinine (SCr), and hematuria were measured weekly. Staining for iron, trichrome, and EMT (vimentin, E-cadherin, smooth muscle actin) markers was performed on the remnant kidneys. ROS were detected in the kidneys by protein carbonyl assay and immunohistochemistry for heme oxygenase 1 (HMOX1), at the end of the study. Apoptosis was detected by TUNEL assay. Warfarin treatment resulted in a PT increase 1.5–2.5 times from control and an increase in hematuria and SCr. Histologically, warfarin-treated animals had more iron-positive tubular epithelial cells and increased IFTA as compared to control (42.9 ± 17% vs. 18.3 ± 2.6%). ROS were increased in the kidney in warfarin-treated rats. The number of tubules that show evidence of EMT was significantly higher in warfarin-treated 5/6NE as compared to control 5/6NE rats. The number of apoptotic tubular epithelial cells was higher in warfarin-treated 5/6 NE rats. Chronic hematuria results in increased iron-positive tubular epithelial cells, EMT, apoptosis, and more prominent IFTA in CKD rats. Our data suggest an important role of chronic hematuria in the progression of CKD.

## Translational Statement

These data demonstrate that hematuria accelerates the progression of chronic kidney injury by increasing interstitial fibrosis and inducing EMT. Considering that many patients with kidney diseases have chronic hematuria, this novel observation suggests new pathogenetic mechanisms of interstitial fibrosis in humans. Translation of our observations to human CKD will allow the development of new therapeutic strategies to decrease the formation of interstitial fibrosis and increase dialysis-free time for patients with chronic kidney insufficiency.

## Introduction

Chronic kidney disease (CKD) is a significant health burden that is associated with premature mortality, decreased quality of life, and increased healthcare expenditures. CKD affects as much as 16% of adult population in the United States (1, 2). It is estimated that the worldwide death rate from CKD is 11.1 per 100,000 population. CKD is ranked number 16 as the leading cause of death in North America (3). There is no effective treatment for CKD; patients ultimately will need kidney replacement therapy such as dialysis or transplantation. The most common etiologies of CKD are glomerular, vascular, and tubulointerstitial diseases that lead to increased interstitial fibrosis and tubular atrophy (IFTA). However, the pathogenesis of IFTA formation is not fully understood. In humans, many kidney disorders are characterized by hematuria and exposure of the tubular epithelial cells to free hemoglobin and iron. Some patients, such as those with IgA nephropathy and thin basement membrane disease, have chronic microscopic hematuria, and some diseases, such as crescentic and necrotizing glomerulonephritis, are accompanied by short-lived but large hematuria. It is not known whether chronic microscopic hematuria is more harmful to the kidney than a shorter but more pronounced hematuria. Recently, emerging evidence indicates an important role of hemoglobin and free iron in the progression of CKD. Free hemoglobin that is released by RBC in the tubular lumen may affect tubular epithelial cells by generating reactive oxygen species (ROS) and increased lipid peroxidation (4). We have previously demonstrated an important role of oxidative stress in the pathogenesis of acute kidney injury (AKI) associated with acute glomerular hemorrhage (5). Epithelial–mesenchymal transition (EMT) of the tubular epithelial cells plays an important role in the progression of IFTA (6, 7), and ROS has been shown to increase EMT *in vivo* (8) and *in vitro* (9, 10). The 5/6 nephrectomy (5/6NE) model is a widely accepted model of CKD, and it is characterized by progressive glomerular injury and increased IFTA (11–13). Using this model, we showed in previous studies that a single episode of large hematuria does not affect the progression of CKD (14).

The aim of the current study was to investigate the associations between chronic hematuria and progression of CKD in 5/6 nephrectomized rats.

## Materials and Methods

These studies have been approved by the Institutional Animal Care and Use Committees (IACUC) at the Ohio State University.

The 5/6 NE was performed in male Sprague Dawley rats (120–140 gm, the Charles River Laboratories, Wilmington, MA) under isoflurane/oxygen (1:5) anesthesia. A nephrectomy of the right kidney and resection of two-thirds of the left kidney were performed simultaneously, as we previously described (14, 15). Hemostasis was achieved by hemostatic sponges (Quick clot; Z-Medica Corporation, Wallingford, CT). The middle laparotomy incision was closed with a 4.0 proline, and the animals were kept at 12h/12h light/dark cycle on the standard rodent diet with free access to water. Warfarin (Millipore Sigma, St. Louis, MO; catalog# PHR1435) was given *per os* in drinking water in the dose of 0.5 mg/kg/day starting 3 weeks after 5/6 nephrectomy to allow recovery from the surgery and wound healing in the remnant kidney. The water consumption per each rat was measured, and the warfarin dose was calculated based on the animal's weight. The warfarin dose was selected based on our previous data (14) and pilot studies to increase PT between 1.5 and 3 times, to mimic the human therapeutic protocol. The control group included 5/6 NE rats that were kept on water. Animals were monitored for 26 weeks after the surgery.

Due to unforeseen restrictions associated with the COVID-19 pandemic, we were able to collect only urine samples on a weekly basis after we initiated the warfarin treatment. Hematuria was measured by Siemens Multistix 5 (Siemens Healthcare Diagnostics Inc, Tarrytown, NY) and expressed in a semi-quantitative scale from 0 to 3, where score 0 is absent, 1+ is trace, 2+ is moderate, and 3+ is large. After the pandemic restrictions were lifted, weekly collection of the blood samples to measure SCr and PT was restored from postoperative week 13 until the end of the study. Blood samples (100 mcl) were collected *via* a tail puncture, as described earlier (5, 14). The animals were sacrificed at week 26 after 5/6 NE; the remnant kidney was dissected for histological and other studies. Histology of the kidney was evaluated on 2- to 3-mcm sections of paraffin-embedded tissue stained with hematoxylin and eosin (H&E), Prussian blue (iron), and trichrome stains. Iron staining was quantitated using a semi-quantitative scale, where 0, negative; 1, mild (involved <25% of the renal cortex and <25% of cells in the cross section of the tubule); 2, moderate (involved between 25–50% of the renal cortex and between 25 and 50% of the cells in the cross section of the tubule); and 3. severe (staining was seen in over 50% of the renal cortex and over 50% of cells in the cross section of the tubule). The scarred areas of the kidney related to the surgical procedure were excluded.

Immunoperoxidase stainings with antibodies to vimentin (Santa Cruz, Dallas, TX cat # SC-373717 1:100), smooth muscle actin (SMA) (Sigma Millipore, Burlington, MA, cat # CBL171 1:50), E-cadherin (BD Bioscience, San Jose, CA, cat # 610181 1:25), and heme oxygenase 1 (HMOX1) (ThermoFisher Scientific, Waltham, MA cat # PA5-27338) were performed on the sections of paraffin-embedded tissue after antigen retrieval [pressure cooker for 1 h with Dako target retrieval solution (Agilent technologies, Santa Clara, CA, cat# S1699)]. Subsequently, the slides were incubated in PBS for 5 min followed by 3% H_2_O_2_ for 5 min. Next, the tissue was blocked with 10% normal goat serum for 10 min. Incubation with primary antibodies was performed for 1 h at room temperature, after which tissue was rinsed with PBS and incubated with the secondary antibody (Santa Cruz, cat # SC-516102 1:100) for 30 min at room temperature. This was followed with PBS wash. Substrate DAB (Vector laboratories, Burlingame, CA, cat#: SK-4105) was added, incubated for 5 min, rinsed with PBS, and rinsed again with water. Slides were counterstained with hematoxylin, treated with blue ammonia water, and cover slipped.

Apoptosis was detected by using a TUNEL Assay Kit—HRP-DAB (Abcam, Waltham, MA, cat # ab206386) based on the manufacturer's protocol. The percentage of the tubules that had positive tubular epithelial cells lining the tubules was evaluated.

Serum creatinine was measured based on the Jaffe reaction using a creatinine reagent assay kit (Raichem, San Marcos, CA) according to the manufacturer's protocol. Briefly, 10 mcl of serum was mixed with 200 mcl of working reagent at 37°C in a 96-well plate, and the absorbance was read at 510 nm at 60 and 120 s on a microplate reader (Molecular Devices, Sunnyvale, CA).

Prothrombin time was measured by using a Fisher Scientific Thromboscreen 200 Hemostasis Analyzer (Fisher Scientific, Middletown, VA) according to the manufacturer's protocol. Briefly, blood was collected into a tube containing 3.8% sodium citrate as the anticoagulant with a ratio of 9:1. The blood was centrifuged at 1,850 RCF for 15 min, 0.05 ml of plasma was placed in the incubation station for 2 min, 0.1 ml of warm thromboplastin was added, and the clotting time was recorded.

We used a “surrogate” INR (sINR) by comparing PT after and before the treatment, as described previously (14, 15). The average PT in 25 rats prior to 5/6 NE was used as the normal PT time (18.2 s).

Reactive oxygen species were analyzed in the renal cortex by using the Protein Carbonyl Assay kit (Cayman Chemical Company, MI) (16) based on the manufacturer's protocol. Briefly, 250 mg of renal cortex was homogenized in ice-cold 2-(N-morpholino) ethanesulfonic acid (MES) buffer (pH 6.7) and centrifuged at 10,000 x g for 15 min at 4°C. Then 0.8 ml of 2,4-dinitrophenylhydrazine (DNPH) was added to the supernatant. For control samples, 0.8 ml of 2.5 M HCl was added. All samples were incubated in the dark at room temperature for 1 h. The samples were precipitated first with 1 mL of 20% trichloroacetic acid (TCA) followed by 10% TCA, and centrifuged at 10,000 g for 10 min. The pellet was washed thrice with 1 mL of ethanol-ethyl acetate (1:1;v/v) to remove free DNPH reagent and centrifuged for 10 min at 10,000 g. The protein pellet was resuspended in 0.5 mL of guanidine hydrochloride with vortexing. Samples were then centrifuged at 10,000 x g for 10 min at 4°C. The concentration of DNPH in the supernatant was determined spectrophotometrically at 370 nm (Versa Max, Molecular Devices), and the molar absorption coefficient of 22, 000 M^−1^ cm^−1^ was used to quantify the levels of protein carbonyls. Protein carbonyl concentration was determined in the samples by the equation: Protein carbonyl (nmol/ml) = (CA)/(^*^0.011μM-1)(500/200 μl), where CA is the corrected absorbance of the samples. Protein carbonyl concentration was calculated per gm of wet tissue.

Kidney injury molecule 1 (KIM-1) levels in the urine were determined by using a quantikine immunoassay ELISA kit (R&D systems, Minneapolis, MN) based on the manufacturer's protocol. Briefly, urine was diluted 1:2, 50 mcl of assay diluent RD1W was added to the microplate well followed by 50 mcl of a sample or standard, and the sample was incubated at room temperature for 2 h. Each well was washed five times with the wash buffer, and 100 mcl of rat KIM-1 conjugate was added to each well and incubated for 2 h at room temperature. Each well was washed five times with the wash buffer, and 100 mcl of substrate solution was added to each well and incubated for 30 min at room temperature. Subsequently, 100 mcl of the stop solution was added to each well, and the optical density of each well was determined at 540 and 450 nm by using the microplate reader (Molecular Devices, Sunnyvale, CA). The reading at 540 nm was subtracted from the reading at 450 nm, and the KIM-1 concentration was calculated based on the standard curve.

### Statistical Analysis

Results are presented as mean ± standard deviation (SD) if not otherwise specified. Differences between two groups were analyzed by the two-paired Student *t*-test or two-way ANOVA test, where applicable.

## Results

### Warfarin Treatment and PT, SCr and Hematuria in 5/6 Nephrectomy Rats

**Figure 1:**
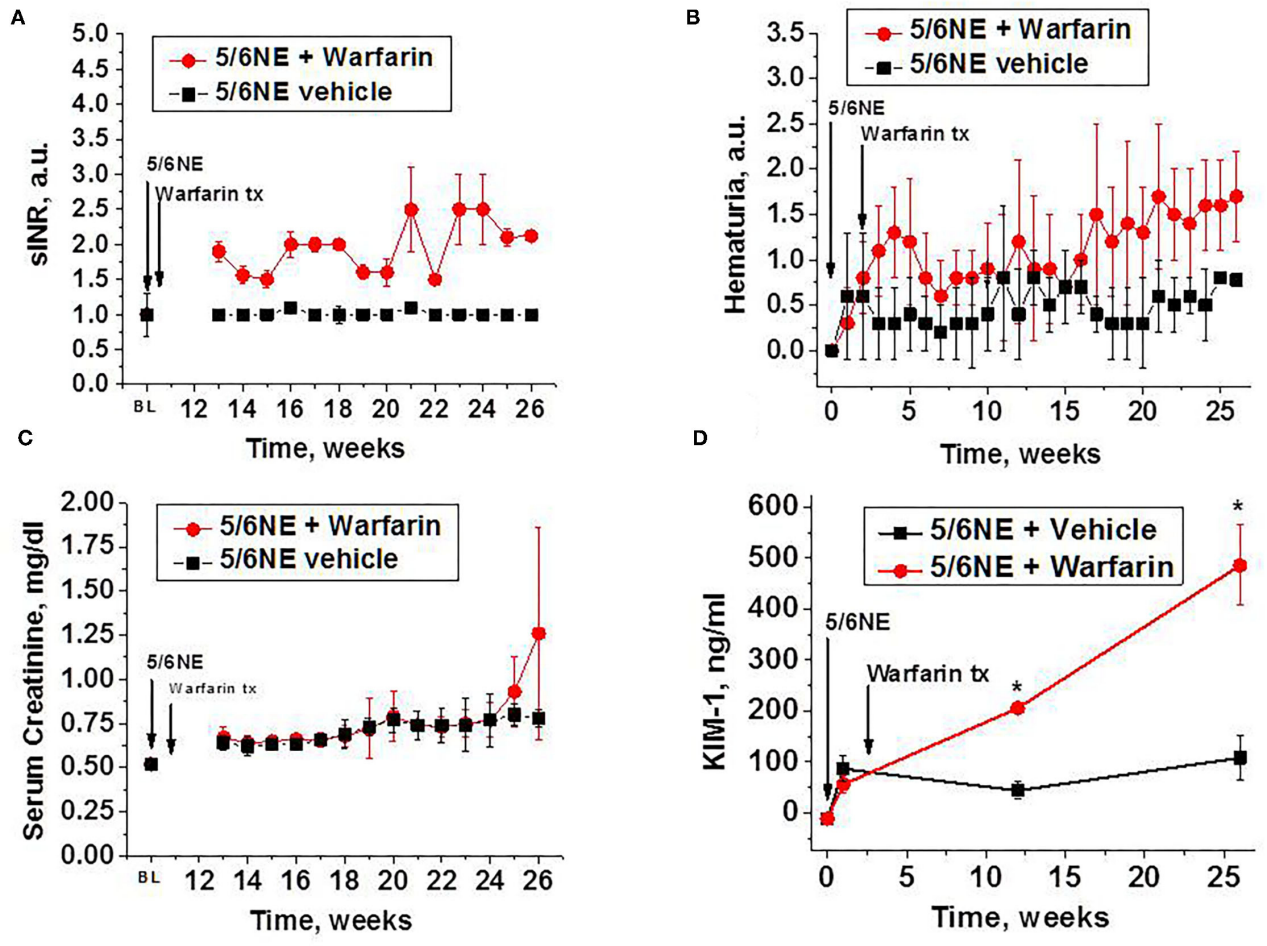
Changes in prothrombin time (PT), hematuria, serum creatinine, and urine kidney injury molecule 1 (KIM-1) in 5/6 nephrectomy rats with and without warfarin treatment. **(A)** Changes in PT in 5/6 nephrectomy rats treated with 0.5 mg/kg/day warfarin (*n* = 7) or vehicle (water, *n* = 6). PT is shown as sINR (time changes from pooled PT in rats prior to the surgery). The 5/6 nephrectomy (5/6NE) and beginning of the treatment with warfarin (warfarin tx) are shown by corresponding arrows. PT was measured prior to the surgery (BL) and weekly starting at week 13 after the surgery. **(B)** Hematuria in 5/6 nephrectomy rats with (*n* = 7) and without (*n* = 6) warfarin treatment. Hematuria is quantitated on the semi-quantitative scale from 0 to 3, where score 0 is absent, 1+ is trace, 2+ is moderate, and 3+ is large. The 5/6 nephrectomy (5/6NE) and beginning of the treatment with warfarin (warfarin tx) are shown by corresponding arrows. **(C)** Serum creatinine levels in 5/6 nephrectomy rats with (*n* = 7) and without (*n* = 6) warfarin treatment. The 5/6 nephrectomy (5/6NE) and beginning of the treatment with warfarin (warfarin tx) are shown by corresponding arrows. Serum creatinine was measured prior to the surgery (BL) and weekly starting at week 13 after the surgery. **(D)** Urine KIM-1 levels in 5/6 nephrectomy rats with and without warfarin treatment. The 5/6 nephrectomy (5/6NE) and beginning of the treatment with warfarin (warfarin tx) are shown by corresponding arrows. BL, baseline (prior to the surgery). *p < 0.05 as compared to control (vehicle-treated) 5/6 nephrectomy rats.

Oral warfarin treatment (0.5 mg/kg/day) increased PT in 5/6 NE rats 1.5–2.5 times from baseline (measured as sINR), while PT in 5/6 NE rats on water (control group) was similar to baseline for the duration of the studies (Figure 1A). Rats treated with warfarin had moderate hematuria for the duration of the warfarin treatment, whereas 5/6 NE control rats had only mild hematuria (two-way ANOVA, *p* = 0.0023) (Figure 1B). Throughout the study period, both groups experienced a steady rise in SCr, but the elevation was higher in 5/6 NE rats treated with warfarin compared to the control group on water. This finding was especially pronounced toward the end of the study (two-way ANOVA, *p* = 0.0043) (Figure 1C). Urine KIM-1 levels were elevated from baseline in all 5/6 NE rats 1 week after the surgery. In control 5/6 NE rats, urine KIM-1 levels were stable for the duration of the study. By contrast, in 5/6 NE rats treated with warfarin, urine KIM-1 levels increased as the study progressed and reached 485.9 ± 78.8 ng/ml at week 26 as compared to 108.1 ± 42.6 ng/ml in control group (*p* < 0.0001, Figure 1D).

### Warfarin Treatment and Histologic Changes in 5/6 Nephrectomy Rats

**Figure 2:**
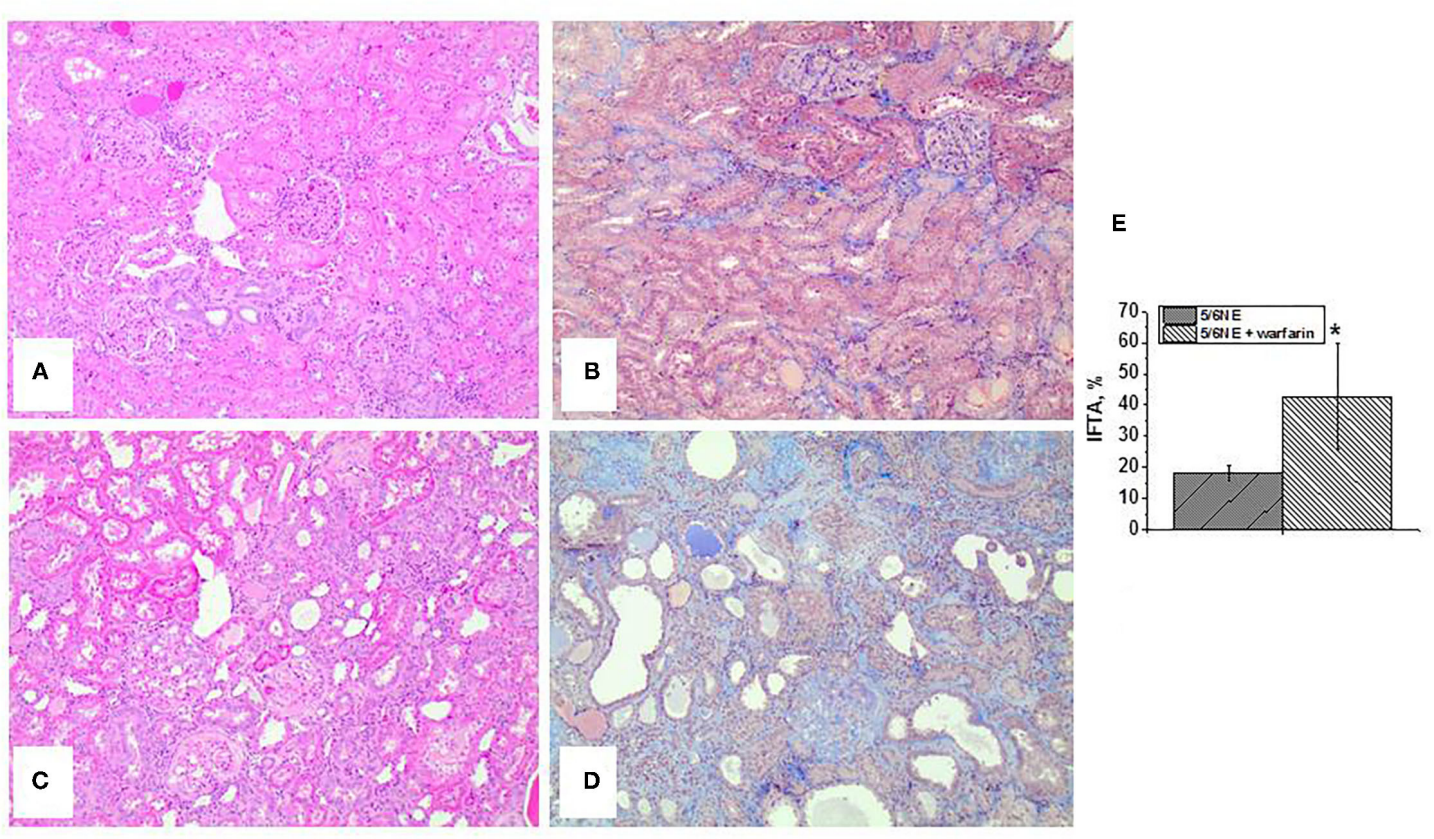
Histologic findings in 5/6 nephrectomy rats with and without warfarin treatment at 26 weeks after the surgery. **(A,B)** Representative images of the renal cortex from a control 5/6 nephrectomy rat stained with hematoxylin and eosin **(A)** and trichrome stain **(B)**. Magnification 100x. **(C,D)** Representative images of the renal cortex from a 5/6 nephrectomy rat treated with 0.5 mg/kg/day warfarin. Sections are stained with hematoxylin and eosin **(C)** and trichrome stain **(D)**. Magnification 100x. **(E)** Quantitative analysis of the degree of interstitial fibrosis and tubular atrophy (IFTA) evaluated as percentage of the renal cortex in 5/6 nephrectomy rats with (*n* = 7) and without (*n* = 6) warfarin treatment. *, *p* < 0.05 compared to control (vehicle-treated) 5/6 nephrectomy rats.

Control 5/6 NE rats had mild (<25%) IFTA, as determined based on H&E and trichrome stains (Figures 2A,B). However, 5/6 NE rats treated with warfarin had moderate-to-severe IFTA (Figures 2C,D). The increase in IFTA was significant (42.86 ± 17.0% in 5/6 NE warfarin-treated rats vs. 18.3 ± 2.6% in control 5/6 NE rats, *p* = 0.0053, Figure 2E).

**Figure 3:**
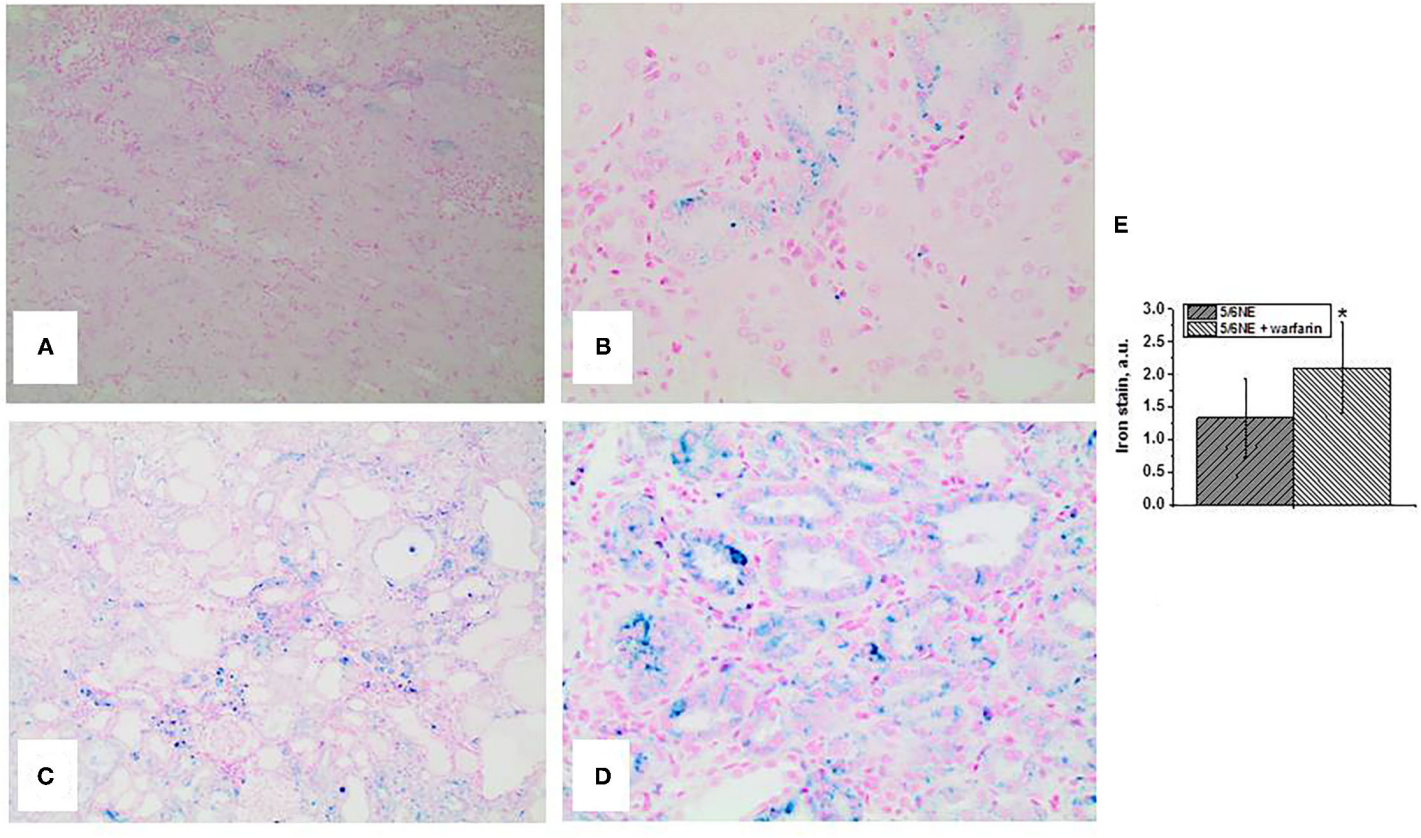
Iron stain in the kidneys in 5/6 nephrectomy rats with and without warfarin treatment at 26 weeks after the surgery. **(A,B)** Iron (Prussian Blue) staining of the renal cortex from a control 5/6 nephrectomy rat. Rare tubules show positive stain, **(A)** magnification 100x. Not all tubular epithelial cells in the cross section of the tubule had positive staining, **(B)** magnification 400x. **(C,D)** Iron (Prussian Blue) staining of the renal cortex from a 5/6 nephrectomy rat treated with 0.5 mg/kg/day warfarin. Many tubules show positive stain, **(A)** magnification 100x. Almost all tubular epithelial cells in the cross-section of the tubule had positive staining, **(B)** magnification 400x. **(E)** Quantitative analysis of the iron stain positivity in the tubules in 5/6 nephrectomy rats with (*n* = 7) and without (*n* = 6) warfarin treatment. Iron staining was quantitated using a semi-quantitative scale, where 0, negative; 1, mild (involved <25% of the renal cortex and <25% of cells in the cross section of the tubule); 2, moderate (involved between 25 and 50% of the renal cortex and between 25 and 50% of the cells in the cross section of the tubule); and 3, severe (lesions are seen in over 50% of the renal cortex and over 50% of cells in the cross section of the tubule). *, *p* < 0.005 compared to control (non-treated) 5/6 nephrectomy rats.

Both control and warfarin-treated 5/6 NE rats had positive staining for iron in the tubular epithelial cells. In control 5/6 NE rats, iron-positive cells were seen in <25% of the tubules (mild) and not in all tubular epithelial cells across the tubule section (Figures 3A,B). In warfarin-treated 5/6 NE rats, iron staining was positive in over 50% of proximal tubular epithelial cells, and almost all the cells in a cross section of the tubule had positive staining for iron (Figures 3C,D). Using a semi-quantitative scale from 0 to 3, the iron positivity in 5/6NE rats treated with warfarin was significantly higher than in 5/6NE vehicle-treated rats (Figure 3E).

**Figure 4:**
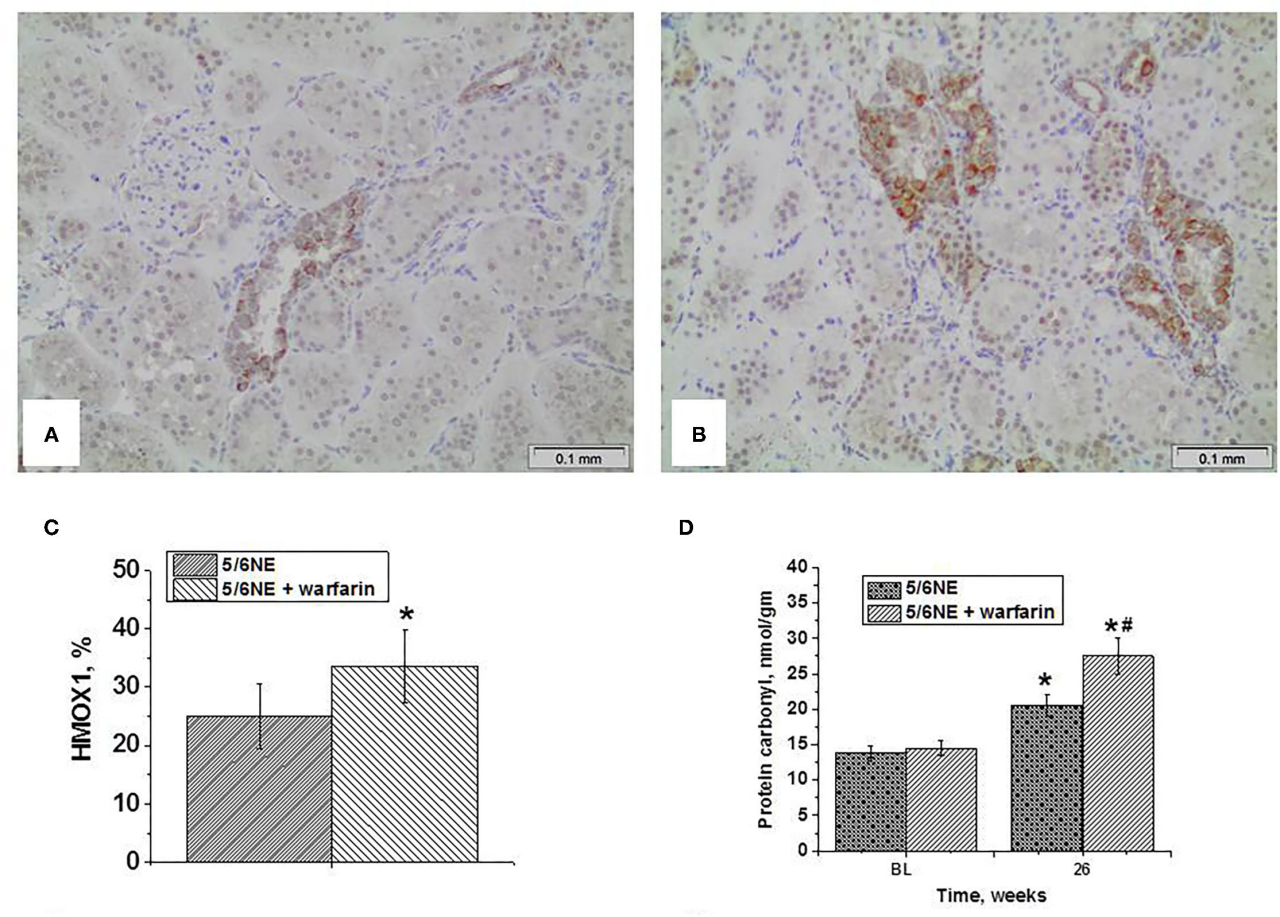
Expression of heme oxygenase 1 in the kidney and protein carbonyl contents in the renal cortex in kidneys in 5/6 nephrectomy rats with and without warfarin treatment before and at 26 weeks after the surgery. **(A)** A representative image of the renal cortex from a control 5/6 nephrectomy rat stained an antibody to heme oxygenase 1 (HMOX1). Immunohistochemistry, Magnification 200x. **(B)** A representative image of the renal cortex from a warfarin-treated 5/6 nephrectomy rat stained an antibody to heme oxygenase 1 (HMOX1). Immunohistochemistry, magnification 200x. **(C)** Quantitative analysis of HMOX1-positive staining in the tubules, percentage (*n* = 6 in 5/6NE control; *n* = 7 in 5/6NE + warfarin groups). **(D)** Protein carbonyls were measured in the renal cortex in the right nephrectomy kidney at the time of 5/6 nephrectomy surgery and in the remnant kidney 26 weeks after the surgery. *, *p* < 0.005 as compared to protein carbonyl contents prior to the surgery; #, *p* < 0.005 as compared to control 5/6 nephrectomy rats.

An increase in iron-positive tubular epithelial cells was accompanied by increased ROS in the kidney as quantitated by protein carbonyl groups. In both control and warfarin-treated 5/6 NE, protein carbonyl groups increased by week 26 after 5/6 NE from baseline (in the right kidneys that were removed at the surgery) (Figure 4D). Elevation in the warfarin-treated group was higher than in control group. Thus, in warfarin-treated 5/6 NE rats, protein carbonyl increased from 14.5 ± 1.1 nmol/gm to 27.5 ± 2.5 nmol/gm (p <0.0001). By contrast, in control rats, this increase was from 13.8 ± 1.1 nmol/gm to 20.5 ± 1.4 nmol/gm (*p* < 0.0001; *p* < 0.0001 between control and warfarin-treated 5/6 NE rats at 26 weeks).

Heme oxygenase 1 expression in the kidney as a marker of oxidative stress was detected by immunohistochemistry. In 5/6 NE rats at 26 weeks, there was an expression of HMOX1 in about 25% of the tubules (Figure 4A), whereas in 5/6 NE rats treated with warfarin, the percentage of HMOX1-positive tubules increased to 33.6% (Figures 4B,C).

### Markers of EMT in the Kidney

**Figure 5:**
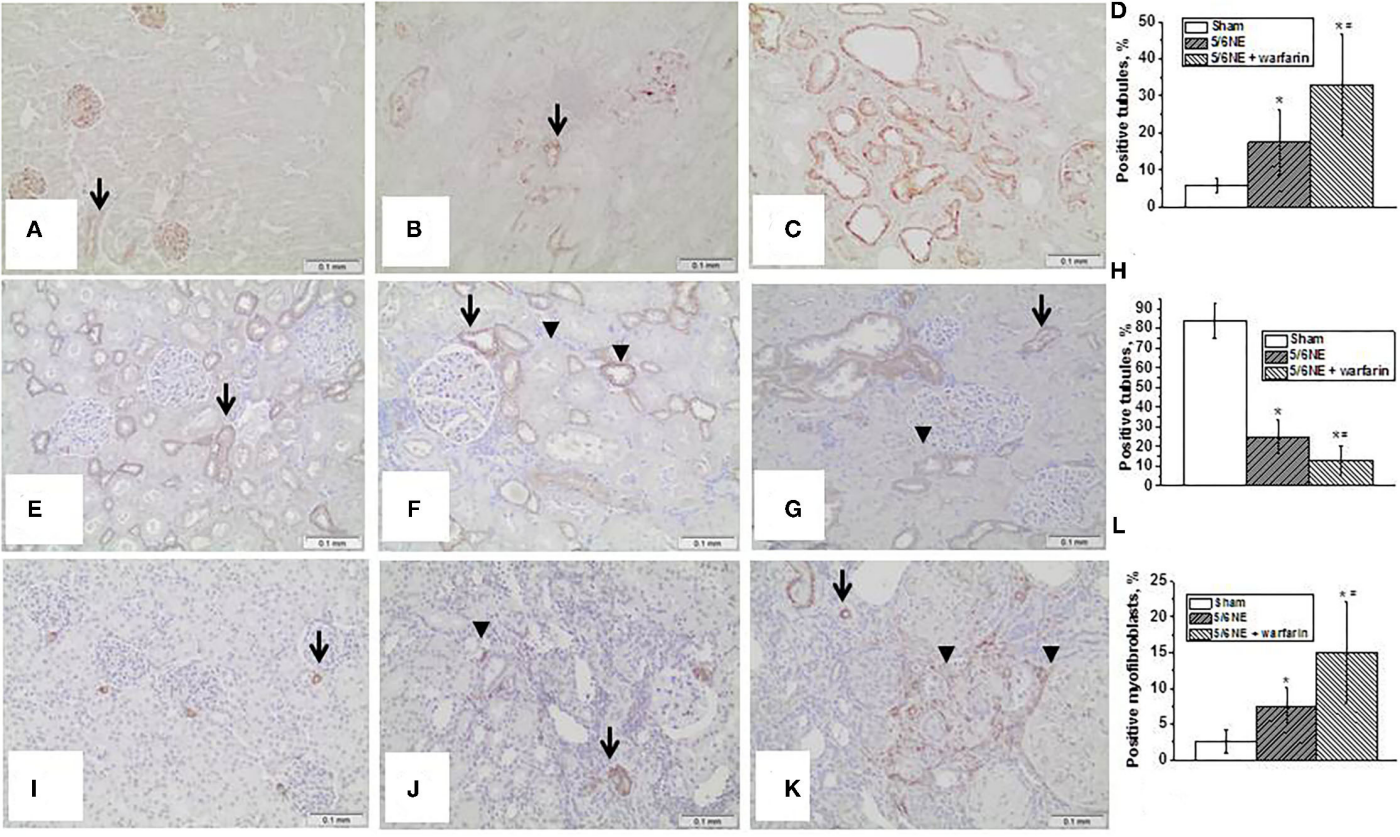
Markers of epithelial–mesenchymal transition in the kidney. **(A–D)** Changes in vimentin expression in the kidney in rats with 5/6 nephrectomy (5/6NE). **(A)** Vimentin staining is positive in the podocytes and endothelial cells in the kidneys prior to 5/6 nephrectomy (arrow), immunohistochemistry, magnification 200x. **(B)** Vimentin staining is positive in some tubular epithelial cells in control 5/6 nephrectomy rats 26 weeks after the surgery (arrow), immunohistochemistry, magnification 200x. **(C)** Vimentin staining is positive in many tubular epithelial cells in 5/6 nephrectomy rats treated with warfarin 26 weeks after the surgery, immunohistochemistry, magnification 200x. **(D)** Quantitative analysis of vimentin-positive staining in the tubules, percentage (*n* = 5 in sham-operated rats; *n* = 6 in 5/6NE control; *n* = 7 in 5/6NE + warfarin groups). **(E–H)** Changes in e-cadherin expression in the kidney in rats with 5/6 nephrectomy. **(E)** Strong linear membranous staining was seen in the distal tubular epithelial cells in the kidneys prior to 5/6 NE, more prominent in the basal surface (arrow), immunohistochemistry, magnification 200x. **(F)** Some distal tubules in control 5/6 nephrectomy rats 26 weeks after the surgery loss strong basal linear membranous staining in the distal tubular epithelial cells (arrowhead), while such pattern was preserved in other tubules (arrow), immunohistochemistry, magnification 200x. **(G)** Almost all distal tubules in 5/6 nephrectomy rats treated with warfarin 26 weeks after the surgery lost strong basal linear membranous staining in the distal tubular epithelial cells (arrowhead), while such pattern was preserved in occasional tubules (arrow), immunohistochemistry, magnification 200x. **(H)** Quantitative analysis of e-cadherin basal-positive staining in the tubules, percentage (*n* = 5 in sham-operated rats; *n* = 6 in 5/6NE control; *n* = 7 in 5/6NE + warfarin groups). **(I–L)** Changes in smooth muscle actin (SMA) expression in the kidney in rats with 5/6 nephrectomy. **(I)** Positive staining for SMA was seen in arteries/arterioles in in the kidneys prior to 5/6 nephrectomy (arrow), immunohistochemistry, magnification 200x. **(J)** In control 5/6 nephrectomy rats 26 weeks after the surgery, positive staining for SMA was seen in not only arteries/arterioles (arrow) but also in occasional interstitial myofibroblasts (arrowhead), immunohistochemistry, magnification 200x. **(K)** In 5/6 nephrectomy rats treated with warfarin 26 weeks after the surgery, positive staining for SMA was seen in not only arteries/arterioles (arrow) but also in many interstitial myofibroblasts (arrowhead), immunohistochemistry, magnification 200x. **(L)** Quantitative analysis of SMA-positive myofibroblasts, percentage of the renal cortex (*n* = 5 in sham-operated rats; *n* = 6 in 5/6NE control; *n* = 7 in 5/6NE + warfarin groups). *, *p* < 0.05 as compared to sham-operated rats; #, *p* < 0.05 as compared to 5/6NE control.

Epithelial–mesenchymal transition in the kidney was evaluated by an increased expression of vimentin in the tubular epithelial cells and a loss of expression of E-cadherin. There was little vimentin expression in the tubular epithelial cells in the kidneys at the time of 5/6 NE (positive staining was seen in podocytes and endothelium, Figure 5A). In control 5/6 NE rats, <10% of the tubules showed positive expression of vimentin in tubular epithelial cells by week 26 (Figure 5B). In 5/6 NE rats treated with warfarin, by week 26 there was a significantly increased percentage of the tubules with positive expression of vimentin in the tubular epithelial cells (35.7 ± 22.0% vs. 8.22 ± 2.3% in control, *p* = 0.0143) (Figure 5C). Quantitative data for vimentin staining in the kidneys are shown in Figure 5D.

Seen synchronously with the changes in vimentin expression, changes were also seen in e-cadherin expression, which in rats is normal in the distal tubular epithelial cells (17). There was membranous staining in the distal tubular epithelial cells in the kidneys prior to 5/6 NE, and a strong linear staining was seen at the basal surface of the cells (Figure 5E). In control 5/6 NE rats 26 weeks after the surgery, there was a loss of the linear basal staining in some tubules but preserved in other distal tubules (Figure 5F). In 5/6 NE rats that were treated with warfarin, the basal linear staining and linear cell membrane staining was lost in the majority of distal tubular epithelial cells (Figure 5G). Quantitative data for e-cadherin basal staining in the distal tubular epithelial cells are shown in Figure 5H.

Smooth muscle actin staining was seen in only the small arteries/arterioles in the kidneys prior to the surgery (Figure 5I). In control 5/6 NE rats at postoperative week 26, such staining was seen not only in the arteries/arterioles but also in occasional interstitial myofibroblasts (Figure 5J). The number of interstitial myofibroblasts was increased in 5/6 NE rats treated with warfarin at 26 weeks after the surgery (Figure 5K). Quantitative data for SMA-positive myofibroblasts in the kidneys are shown in Figure 5L.

### Warfarin Treatment Increases Apoptosis in the Tubular Epithelial Cells

**Figure 6:**
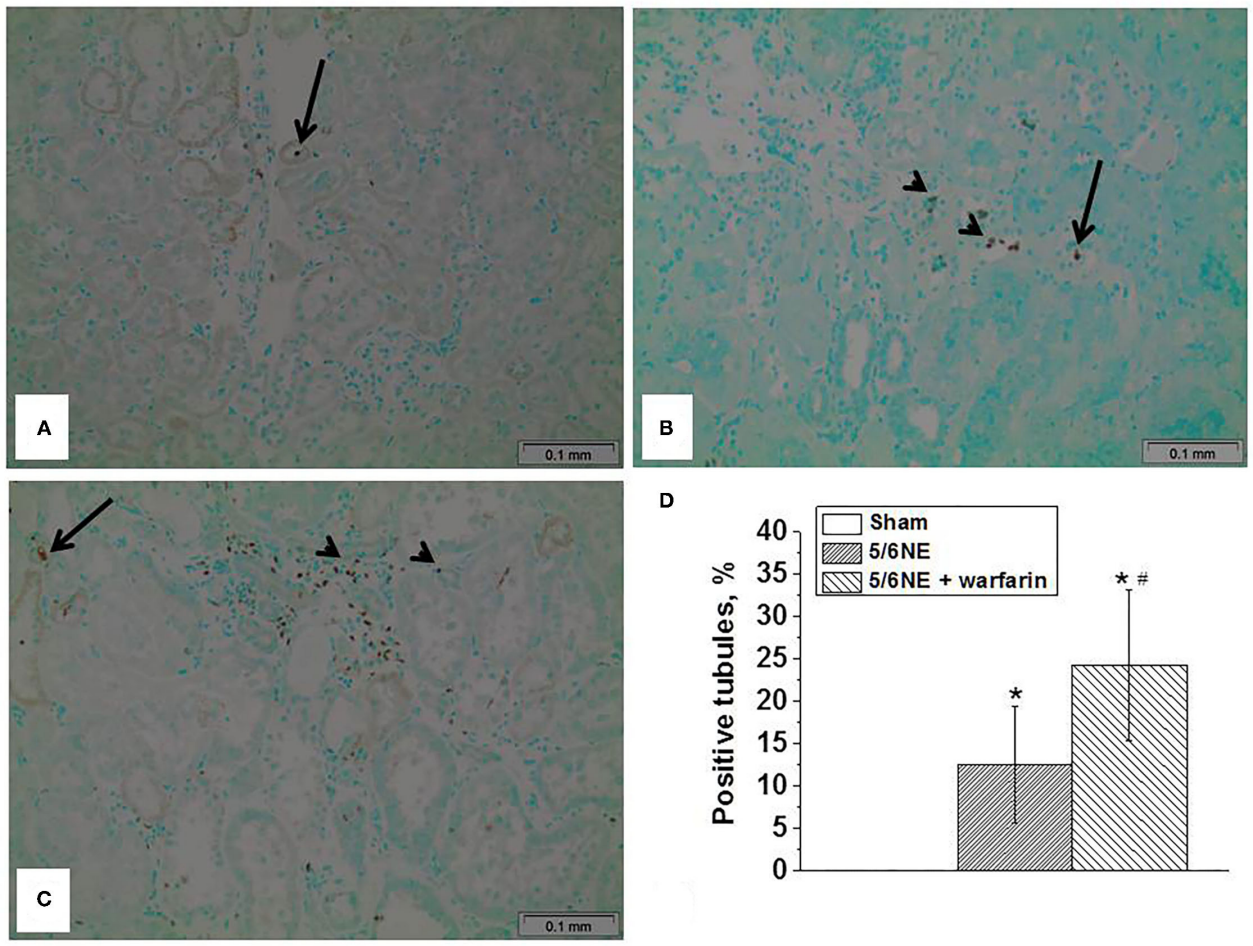
Apoptosis in the tubular epithelial cells in 5/6 nephrectomy rats treated with warfarin. Apoptosis was detected in the sections of paraffin-embedded tissue by using a TUNUEL assay. **(A)** In sham-operated rats, rare sloughed apoptotic cells were seen only in the tubular lumen (arrow), no apoptotic cells were seen in the tubular epithelial cell lining the tubules. **(B)** In 5/6 nephrectomy control rats, apoptotic cells were seen not only in the tubular lumen (arrow), but in the tubular epithelial cells lining the tubules (arrowhead). **(C)** In 5/6 nephrectomy rats treated with warfarin, apoptotic cells were seen not only in the tubular lumen (arrow), but in numerous tubular epithelial cells lining the tubules (arrowhead). **(D)** Quantitative analysis of the percentage of tubules that show apoptotic cells in the tubular epithelial cells lining the tubules (*n* = 5 in sham-operated rats; *n* = 6 in 5/6NE control; *n* = 7 in 5/6NE + warfarin groups). *, *p* < 0.05 as compared to sham-operated rats; #, *p* < 0.05 as compared to 5/6NE control.

Apoptosis was detected in the sections of paraffin-embedded tissue by a TUNEL assay. In sham-operated rats at 26 weeks, there were only occasional sloughed off apoptotic cells in the tubular lumen, but no apoptotic cells were seen in the tubular epithelial cells that cover the tubules (Figure 6A). In 5/6 NE control rats treated with vehicle, at 26 weeks after the surgery, apoptotic cells were seen not only in the tubular lumen, but also in about 12% of the tubules in the tubular epithelial cells that cover the tubules (Figure 6B). Warfarin treatment resulted in an increased number of apoptotic cells in the tubular epithelial cells that cover the tubules. and it was present in 24% of the tubules (Figure 6C).

## Discussion

To the best of our knowledge, this is the first study that describes the effects of long-standing microscopic hematuria on the progression of CKD. CKD is a common outcome of many kidney diseases. CKD may be the result of primary vascular, glomerular, or tubulointerstitial injury. In humans, glomerular injury associated with diabetes mellitus, hypertension, and other glomerulonephritis accounts for ~75% of all cases of CKD in adults (18). Tubulointerstitial diseases are cause of the remainder of CKD cases. Therefore, the two main causes of CKD may be described: (1) vascular/glomerular and (2) tubulointerstitial diseases. All these etiologies have common outcome: increased interstitial fibrosis and progressive decline in kidney function.

Hematuria is a common symptom in human kidney diseases and can be seen in different glomerulonephritis. Hematuria may be seen in diseases associated with immune complex deposition in the glomeruli (IgA nephropathy, lupus nephritis, etc.). In other diseases with abnormalities in the glomerular basement membrane or filtration barrier [thin basement nephropathy, focal segmental glomerular sclerosis (FSGS) etc.], hematuria may be present as well. Human kidney diseases are rarely present with one symptom, and this principle applies to hematuria as well. Another condition is associated proteinuria in addition to hematuria. The role of hematuria in the pathogenesis of interstitial fibrosis is not well elucidated. We had previously demonstrated that treatment with anticoagulants results in hematuria in rats, and a single episode of hematuria does not affect the progression of CKD in 5/6 nephrectomy rats (14). In the current study, we used treatment with warfarin to elevate the PT to 1.5–2.5 times from normal in order to mimic the therapeutic regimen in humans, and this warfarin treatment protocol increased hematuria in rats (Figure 1B). Of note, 5/6 NE control rats (not treated with warfarin) also had microscopic hematuria, but to a lesser degree, which corresponds with earlier experimental data (5, 14).

There are several mechanisms that could explain tubular epithelial cell injury in hematuria. It has been demonstrated that proximal tubular epithelial cells can phagocyte erythrocytes. Using electron microscopy, erythrocytes have been visualized in the phagolysosomes of tubular epithelial cells. The red blood cells show varying stages of degradation, including with collapse and breakdown of the cell membrane and invasion by cytoplasmic organelles (19). In our studies, we identified iron in the tubular epithelial cells in both control and warfarin-treated 5/6 NE rats 26 weeks after the surgery. The number of iron-positive tubular epithelial cells was significantly higher in warfarin-treated rats as compared to the control (Figure 3E). Of note, only long-term hematuria leads to the iron accumulation in the tubular epithelial cells, in our early work we demonstrated that in rats with short-term hematuria (7 days), iron stain is negative in the tubules, even in those with RBC casts (5). These data suggest that chronic hematuria is necessary to accumulate iron in the tubular epithelial cells. Indeed, in a glycerol model of heme protein-induced renal injury, repeated, but not single, insults to the kidney resulted in increased tubulointerstitial injury (20).

Several studies describe toxic effects of heme on cultured tubular epithelial cells *in vitro*. Heme accelerates apoptosis, induces HMOX1-dependent p21 expression, provokes cell cycle arrest, and inhibits cell growth (21). Iron promotes hydroxyl radical formation in the kidney, and treatment with iron chelators protects kidney epithelial cells from ischemia/reperfusion injury (22). Interestingly, chronic administration of angiotensin II to rats results in an increased iron accumulation in the kidney, and the toxic effect of iron on the tubular epithelial cells may be ameliorated byHMOX1 (23, 24). In a case of a patient with HMOX1 deficiency, the patient had persistent proteinuria and hematuria. Morphologic findings in the kidney on biopsies and autopsy showed progressive chronic kidney injury, which included endothelial cell injury, tubulointerstitial injury with tubular dilatation and atrophy, interstitial fibrosis, and inflammatory cell infiltrates. Additionally, there were tubular epithelial cell injury and massive deposition of iron and haptoglobin in the tubular epithelial cells (25). In the current study, we found increased ROS in the renal cortex in both control and warfarin-treated 5/6 NE rats, but a more prominent increase in the group treated with warfarin. The expression of HMOX1 in the kidney was also higher in 5/6 NE rats treated with warfarin as compared to control 5/6 NE rats at 26 weeks (Figure 4). Tubular epithelial cell injury was evident by increased levels of KIM-1 in the urine in 5/6 NE rats treated with warfarin (Figure 1D). We had demonstrated earlier that treatment with ROS chelator N-acetylcysteine (NAC) prevents acute tubular necrosis associated with warfarin-induced hematuria, suggesting an important role of ROS in the pathogenesis of tubular epithelial cell injury (5). Apoptosis of the tubular epithelial cells was more prominent in rats treated with warfarin than in 5/6 NE control rats. The percentage of tubules with apoptotic tubular epithelial cells was twice higher in 5/6 NE rats treated with warfarin as compared to control 5/6 NE rats (Figure 6D). It has been demonstrated that ROS and iron plays a significant role in the apoptosis of tubular epithelial cells, supporting our findings (26).

It appears that hematuria-associated increase in ROS in the kidneys promotes EMT in the tubular epithelial cells in both *in vivo* and *in vitro* (8–10). It has been demonstrated that EMT is increased in patients with IgA nephropathy, a disease characterized by long-standing microscopic hematuria (27). Similarly, our data indicate that in 5/6 NE rats EMT increased with the degree of hematuria. In control 5/6 NE rats that had mild hematuria, <10% of the tubules expressed vimentin (mesenchymal marker), whereas in 5/6 NE rats that were treated with warfarin and had moderate chronic hematuria, over 30% of the tubules expressed vimentin in tubular epithelial cells (Figures 5B,C). There was also a loss of linear staining for e-cadherin in the distal tubules (Figures 5F,G) and increased number of interstitial myofibroblasts (Figures 5J,K), which occurred synchronously with the change in vimentin expression.

Interestingly, SCr levels were not reflective of the degree of chronic kidney injury in experimental animals. Even though SCr levels increased from baseline by week 26 by about 50% in both control and warfarin-treated 5/6 NE rats, and the differences in SCr levels between control and warfarin-treated 5/6 NE rats were statistically significant for the duration of the studies, these SCr levels were not statistically different between these groups at any given week after the surgery (Figure 1C). However, the differences in IFTA between control and warfarin-treated 5/6 NE rats were significant (Figure 2E). Our findings correspond with the clinical observations that SCr is a poor marker of underlying chronic kidney injury. It is not sensitive, and it does not increase until substantial tubulointerstitial injury is developed (28, 29). We observed increasing SCr levels in 5/6 NE rats treated with warfarin compared to control 5/6 NE rats at weeks 25 and 26 (Figure 1C), suggesting that prolonged observations could have yield significant elevations in SCr in warfarin-treated rats.

Limitations of our studies include that only one anticoagulant is tested. However, our previous data indicate that the short-term kidney effects of other anticoagulants (such as direct thrombin inhibitor dabigatran) are similar to those of warfarin (30–33). Studies with other anticoagulant classes (such as direct thrombin inhibitors or Factor X antagonists) are warranted to investigate their effects on kidney function, but based on our data, the main effect of warfarin on the kidney fibrosis is mediated through hematuria; therefore, one may suggest that effects of other anticoagulants will be similar to those of warfarin.

In conclusion, we demonstrated that chronic hematuria results in iron accumulation in the tubular epithelial cells in the kidney, increased ROS in the renal cortex, increased EMT in the tubular epithelial cells, increased apoptosis in the tubular epithelial cells, and increased IFTA. Our findings suggest an important role of chronic hematuria in the progression of CKD.

## Data Availability Statement

The raw data supporting the conclusions of this article will be made available by the authors, without undue reservation.

## Ethics Statement

The animal study was reviewed and approved by IACUC at the OSU.

## Author Contributions

MX and AM: conducted experiments, participated in writing, and editing the manuscript. SB: concept development, the study design, data analysis, writing, and editing the manuscript. LB, AS, II, and BR: participated in the study design, data analysis, writing, and editing the manuscript. All authors contributed to the article and approved the submitted version.

## Funding

This study was were supported by the NIH NIDDK Grant R01DK117102 to SB.

## Conflict of Interest

The authors declare that the research was conducted in the absence of any commercial or financial relationships that could be construed as a potential conflict of interest.

## Publisher's Note

All claims expressed in this article are solely those of the authors and do not necessarily represent those of their affiliated organizations, or those of the publisher, the editors and the reviewers. Any product that may be evaluated in this article, or claim that may be made by its manufacturer, is not guaranteed or endorsed by the publisher.
